# Migrasomes in kidney diseases: emerging molecular mechanisms, diagnostic value, and therapeutic prospects

**DOI:** 10.1042/BST20260952

**Published:** 2026-10-23

**Authors:** Wei Qin, Bing Li, Yixian Zhang, Ping Nie

**Affiliations:** Department of Nephropathy, The Second Hospital of Jilin University, Changchun 130041, Jilin Province, China

**Keywords:** biomarkers, cell migration, molecular mechanisms, renal physiology

## Abstract

Migrasomes are migration-dependent extracellular organelles that form on retraction fibers of migrating cells. Although they can enter the extracellular space, their biogenesis and membrane organization are distinct from those of conventional extracellular vesicle subtypes. They are involved in intercellular signaling, clearance of cellular material, and maintenance of mitochondrial homeostasis. Emerging evidence suggests their involvement in the development of kidney diseases, such as podocyte injury, membranous nephropathy, diabetic kidney disease (DKD), and renal cell carcinoma (RCC). Podocyte-derived migrasomes in urine have shown potential as non-invasive biomarkers for early detection of kidney injury, with levels correlating with PLA2R antigen expression in membranous nephropathy. In DKD, migrasomes may help mitigate oxidative stress by removing damaged mitochondria. In clear cell renal cell carcinoma, they appear to influence the tumor microenvironment, affecting disease progression and prognosis. However, research on migrasomes in kidney diseases is still in its early stages. Challenges, including an incomplete understanding of their mechanisms and a lack of standardized detection methods, limit clinical application. This review outlines recent advances in understanding migrasomes and explores their potential for early diagnosis, disease monitoring, and prognosis evaluation in kidney diseases, while discussing existing barriers to guide future research efforts.

## Introduction

Chronic kidney disease affects approximately 10% of the global population and is characterized by complex pathological processes, including inflammation, oxidative stress, and metabolic dysregulation [1–3]. Despite rapid advances in the field of nephrology, the early diagnosis of kidney disease remains a challenge, necessitating the identification of biomarkers that reflect early-stage damage.

Extracellular vesicles (EVs) contribute to kidney disease as biomarkers [4,5], delivery vehicles [6,7], and mediators of intercellular communication [8,9]. However, conventional EVs are released by many cell types and carry heterogeneous cargo [10], which complicates assignment of disease-specific functions to discrete EV subtypes [4,11,12]. In addition, much of their signaling appears to operate over short distances within tissue microenvironments, limiting their value for delineating system-level mechanisms [13,14].

Migrasomes are recently identified migration-dependent organelles that should be distinguished from conventional EV subtypes rather than simply classified as another EV subtype. They are micron-scale, vesicle-containing structures that arise by localized swelling and budding at the tips or branch points of retraction fibers left behind migrating cells and can become extracellular after retraction-fiber breakage [15]. This biogenetic route differs fundamentally from exosome release through multivesicular-body fusion with the plasma membrane, microvesicle shedding by outward plasma-membrane budding, and apoptotic-body formation during apoptotic blebbing and fragmentation. Migrasome biogenesis is governed by a distinct molecular program involving SMS2/sphingomyelin-dependent nucleation, the PIP5K1A-PI(4,5)P2-Rab35-integrin axis during maturation, and tetraspanin/cholesterol-enriched membrane domains during expansion, rather than the canonical ESCRT-centered machinery associated with many exosomal pathways [16–20]. Their membranes are notably enriched in sphingomyelin and TSPAN4-containing membrane microdomains, which contribute to membrane rigidity and organelle expansion [18–20]. Functionally, migrasomes can carry bioactive cargo, including mRNAs and proteins, and mediate intercellular communication through migracytosis [21,22]. They may also participate in cellular quality control by sequestering damaged mitochondria and other cellular components, a process that has been implicated in renal tubulointerstitial fibrosis and oxidative stress [23,24] (Table 1).

**Table 1:** Comparison of characteristics between migrasomes and other EVs

Vesicle types	Diameter	Morphology	Formation mechanism	Contents	Markers	Functions	Applications in kidney disease
Exosome	30–150 nm [74]	Lipid bilayer vesicle [74]	Derived from the fusion of multivesicular bodies with the plasma membrane, dependent on the ESCRT complex and Alix protein [75]	Proteins associated with the ESCRT complex, such as Alix and TSG101, and specific miRNAs [75]	Tetraspanin superfamily (CD63, CD9, CD81, CD82), TSG101, and Alix [76]	Transmit miRNAs and proteins, mediating intercellular communication [77]	Alleviating oxidative stress [78], apoptosis [79,80], inflammation [81,82], fibrosis [78,79,83] and autophagy [84]
Microvesicles (ectosome)	100–1000 nm [85]	Membrane structure enveloped by a lipid bilayer [86]	Direct outward budding and fission of plasma membrane	Certain organelles and mRNA	Proteins, lipids, cytokines, mRNAs, miRNAs (miRNA-350-3p) [87]	Pro-inflammatory [88]; immunomodulatory [89]	Serve as a biomarker for kidney injury
Apoptotic bodies	500–5000 nm [90]	Membrane-bound vesicles containing fragmented cell components	Produced during apoptosis by membrane blebbing and cell fragmentation	DNA fragments, histones, organelles, proteins, RNAs	Phosphatidylserine, Annexin V binding, cleaved caspase-3;	Induces M2 polarization of macrophages and immune responses [91]	Reflect renal cell apoptosis, serves as an early biomarker for kidney injury [92]
Migrasome	300–3000 nm [93]	Pearl necklace, pomegranate-like [27]	Dependent on cell migration, formed by local membrane protrusions of contractile fibers [94], independent of the ESCRT system [18]	Proteins (such as tetraspanin TSPAN4), RNA, and damaged mitochondria [19,64]	TSPAN4, TSPAN7, NDST1, PIGK, CPQ, EOGT [45]	Migration, mitochondrial extrusion [35]	Serves as an early diagnostic biomarker for podocyte injury [44,46,52]; Regulates clear cell renal cell carcinoma
Large oncosome	1000–10 000 nm [95]	Very large, irregular vesicles [95]	Formed by direct budding of the plasma membrane of tumor cells [95]	MiRNA, typical DNA mutations, regulatory microRNAs and oncoproteins [96]	Tumour-specific EGFRvIII [95]	Mediates signaling between tumor cells [97] and cellular reprogramming [98]	Novel diagnostic and prognostic biomarkers for urologic malignancies [99]

This review focuses on the biological characteristics of migrasomes most relevant to kidney disease, summarizes the current evidence in the field of nephrology, and explores the potential of migrasomes in diagnostics and translational medicine, as well as the major obstacles to their clinical application.

## Biological features of migrasomes relevant to kidney disease

### Discovery and defining characteristics of migrasomes

In 1963, Taylor and Robbins documented elongated tubular structures at the posterior base of migrating cells and designated them as retraction fibers [25]. In 2015, Ma and colleagues identified a previously unreported vesicle-containing structure associated with retraction fibers surrounding rat kidney cells while investigating their motility characteristics. Unlike conventional EVs, these structures contained multiple small vesicles, 50–100 nm in diameter, within their lumen. Owing to their morphological resemblance to an unsealed pomegranate, these entities were provisionally termed “pomegranate-like structures” [15]. Through proteomic analysis and green fluorescent protein tracking, the researchers identified the specific molecular marker of these structures as TSPAN4-a protein with four transmembrane domains. Pharmacological modulation of cell migration altered their abundance, indicating that their formation is closely linked to migratory activity. These structures were therefore named “migrasomes” and are now generally defined as migration-dependent, vesicle-containing organelles that form at the tips or branch points of retraction fibers extending from migrating cells [15].

### Mechanism of migrasome formation

Migrasome biogenesis is a complex biological process that can be divided into three distinct stages: nucleation, maturation, and expansion [17] (Figure 1).

**Figure 1:**
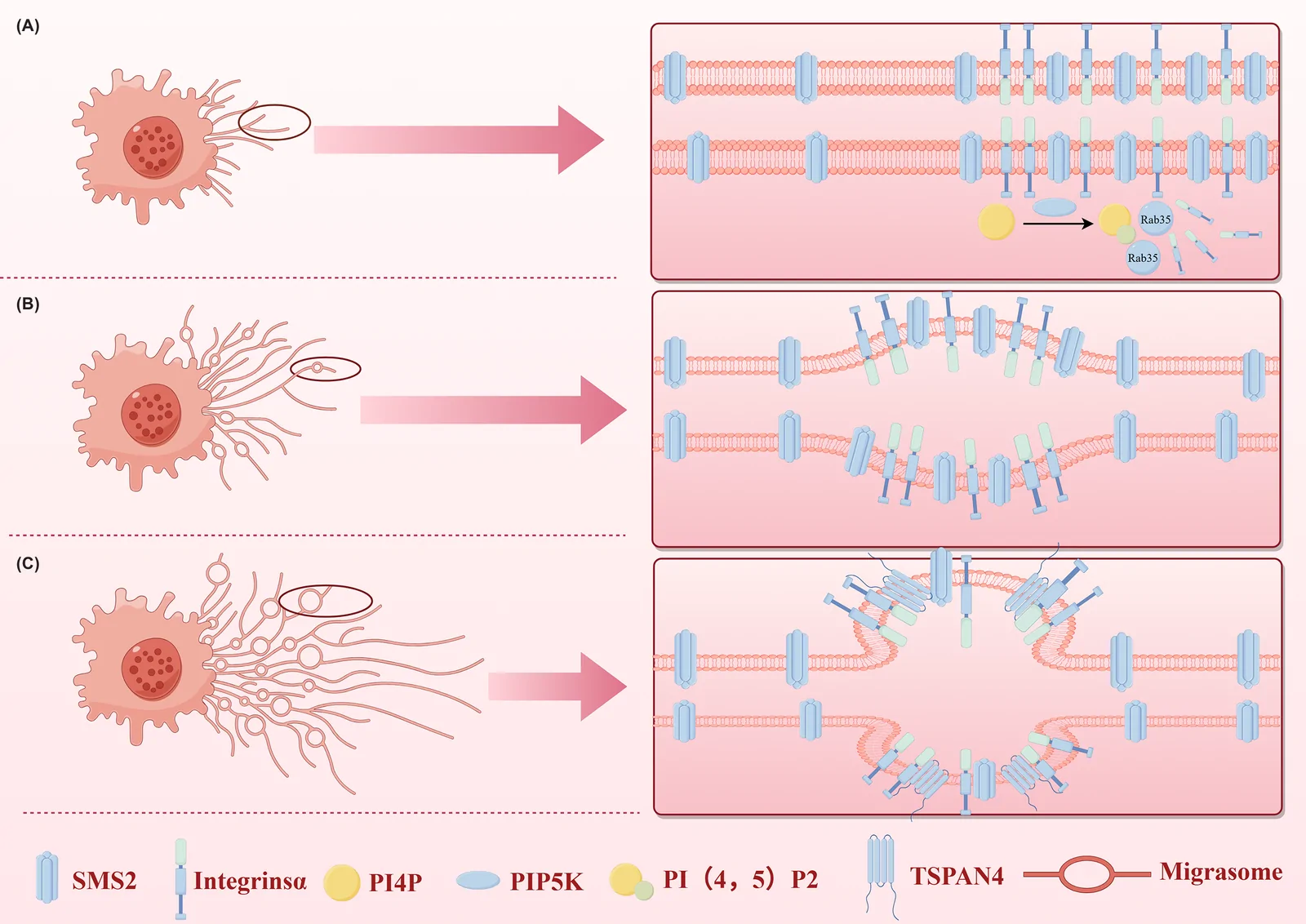
Mechanism of migrasome formation (**A**) Nucleation: Initial clustering of SMS2 at the leading edge of the cell. (**B**) Maturation: Local swelling occurs at nucleation sites along retraction fibers, giving rise to unstable precursor structures. (**C**) Expansion: Tetraspanins and cholesterol form TEMs, which gradually assemble into TEMAs, progressively forming the migrasome. Figure created with Figdraw.

Nucleation involves sphingomyelin synthesis by sphingomyelin synthases (SMSs). SMS1 is exclusively localized to the Golgi apparatus, whereas SMS2 distributes in both the Golgi apparatus and plasma membrane [26]. In migrating cells, SMS2 assembles into stable puncta on the basolateral membrane that anchor to the extracellular matrix (ECM) [27], and these sites frequently seed migrasomes [18]. Knockdown of SMS2 reduces migrasome formation, while exogenous sphingomyelin restores it, indicating that SMS2-dependent sphingomyelin production initiates biogenesis [18].

During maturation, migrasomes undergo pronounced morphological changes characterized by localized swelling of retraction fibers at nucleation sites, resulting in the formation of unstable precursor structures [28]. Integrins accumulate at tips or branch points before migrasome emergence, mediating cell–ECM contacts that support growth [29,30]. The phosphatidylinositol 4-phosphate 5-kinase (PIP5K) signaling pathway regulates the selective recruitment of integrins to sites of chemotaxis. PIP5K1A is recruited to the site of migrasome formation, where it catalyzes the phosphorylation of phosphatidylinositol 4-phosphate to form phosphatidylinositol 4,5-bisphosphate, which in turn recruits the small GTPase Rab35. This process specifically regulates the localization of integrin α5 to this site; thereby, integrins provide an important physical interface between migrating cells and the ECM, anchoring retraction fibers and nascent migrasomes to the underlying ECM. These adhesion sites can serve as mechanical anchoring points, against which forward cell migration generates membrane tension within retraction fibers. Mechanical forces can thereby be converted into membrane-curvature signals. Retraction fibers are highly curved membrane tubules, and TSPAN4, a key structural determinant of migrasome formation, preferentially localizes to membranes with high positive curvature [19,31–33]. Experimental manipulation of membrane tension has shown that increasing membrane tension enhances TSPAN4 enrichment, while quantitative modeling further indicates that TSPAN4 accumulation increases with increasing membrane tension [32]. These findings suggest that the physical state of the membrane itself can directly regulate the spatial organization of migrasome-associated proteins. Subsequently, curvature-sensitive TSPAN4 accumulates in these regions, thereby stabilizing the expanding migrasomes [32].

### Material transport by migrasomes

Migrasomes are membrane-bound structures that contain numerous vesicles and intracellular components. Cells can release intracellular materials, such as red fluorescent protein, through migrasomes [15]. As important carriers of signaling molecules, migrasomes are enriched in a variety of functional proteins, including chemokines and cytokines.

These fluorescently labeled migrasomes can be transferred between cells, suggesting that migrasomes may participate in signal transmission and material exchange, thereby contributing to intercellular communication. These vesicles are subsequently transported to migrasomes and are ultimately released in a directional manner through membrane fusion [34]. The intracellular transport of vesicles within migrasomes involves the coordinated activity of several key proteins. Rab10 and caveolin-1 colocalize within migrasomes, and their transport depends on myosin Va and Rab-interacting lysosomal protein-like 2 [22]. In parallel, Rab8α and Rab11 contribute to vesicle targeting, while SNARE complexes, such as VAMP2 with syntaxin 4 and SNAP23, mediate membrane fusion and vesicular contents release at migrasomes [34]. Through this coordinated system, cytosolic components can be selectively transported and released in a migration-dependent manner, a process termed “migracytosis” [35].

### Biological properties with potential renal relevance

Beyond their structural features, migrasomes participate in several biological processes that may be relevant to renal injury, including intercellular communication, mitochondrial quality control, and microenvironmental remodeling [23] (Figure 2).

**Figure 2:**
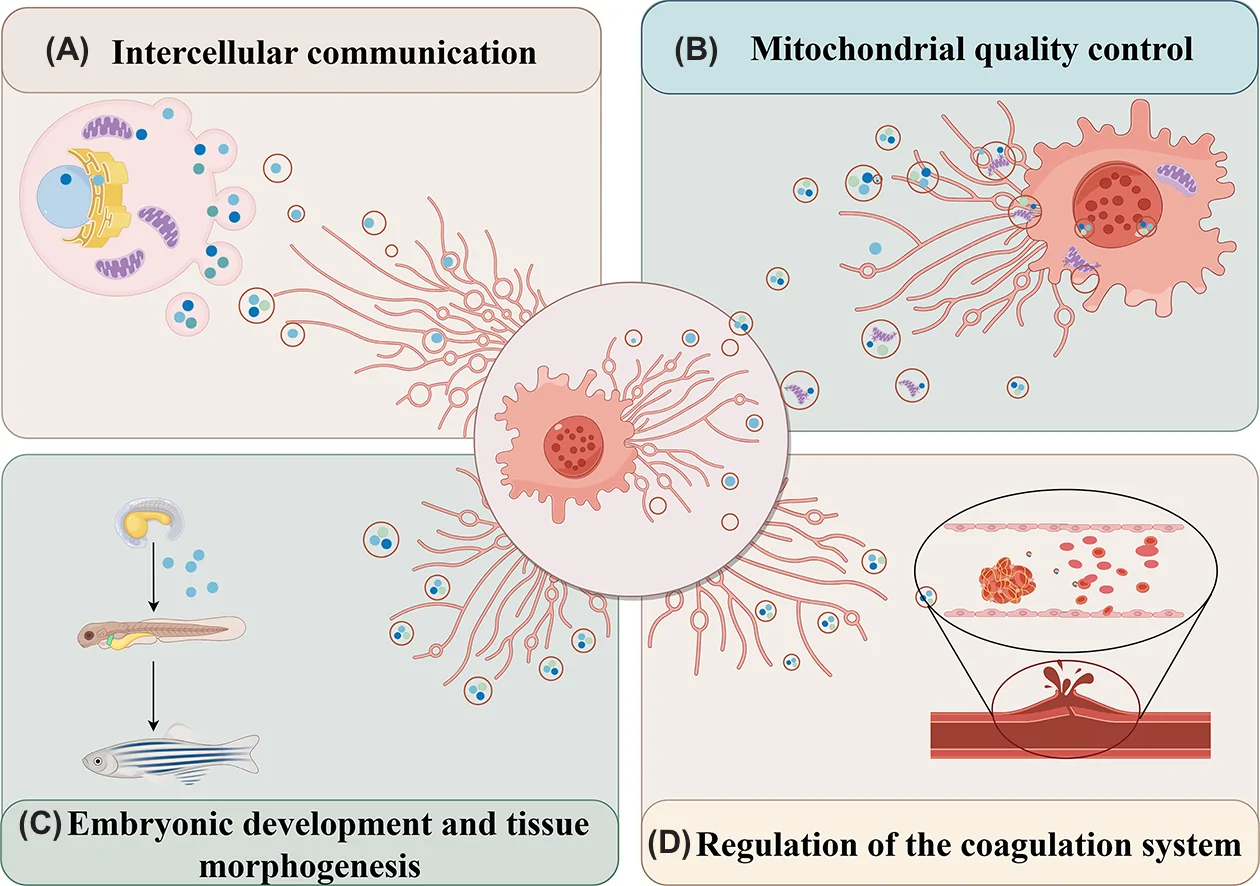
Biological properties with potential renal relevance (**A**) Intercellular communication: migrasomes release internal vesicles and signaling molecules through membrane rupture or leakage, which are then taken up by neighboring cells. (**B**) Mitochondrial quality control: migrasomes can selectively encapsulate dysfunctional mitochondria and expel them from the cell, maintaining mitochondrial homeostasis within the cell. (**C**) Embryonic development and tissue morphogenesis: migrasomes precisely regulate the directional migration of mesodermal cells by enriching chemokines, thereby participating in gastrulation and organ morphogenesis. (**D**) Regulation of the coagulation system: migrasomes may be involved in the composition and regulation of the coagulation system. Figure created with Figdraw.

Migrasomes are released by migrating cells along retraction fibers and can discharge internal vesicles and signaling molecules into the extracellular space, where these contents may be taken up by neighboring cells [36]. Sequencing analyses have shown that migrasomes are enriched in mRNAs related to metabolism, intracellular transport, cell junction formation, vesicle fusion, and membrane organization. Functional studies further demonstrated that migrasome-derived phosphatase and tensin homolog (PTEN) protein and PTEN mRNA can be transferred to PTEN-deficient recipient cells and regulate pAKT signaling with distinct temporal patterns, supporting a role for migrasomes in horizontal cargo transfer [36].

Furthermore, several signaling proteins, including C-X-C motif chemokine ligand 12 (CXCL12), are selectively trafficked to migrasomes through constitutive or regulated secretory pathways, thereby enabling long-distance intercellular communication [34,37]. In addition, migrasomes undergo selective cargo sorting, leading to the enrichment of factors such as CXCL12 and vascular endothelial growth factor A (VEGFA) through a process termed “migracytosis” [35]. Migrasomes also concentrate cytokines such as tumor necrosis factor-α and interleukin-6 (IL-6) at sites of inflammation [38], thereby facilitating rapid responses to changes in the local microenvironment and regulating critical processes such as tissue regeneration and angiogenesis [34,38,39].

Another property of potential renal relevance is mitochondrial quality control. Migrasomes contribute to mitochondrial homeostasis by selectively sequestering and removing dysfunctional mitochondria, such as those with reduced membrane potential or elevated reactive oxygen species, through a process termed “mitocytosis” [40,41]. This migrasome-mediated quality control mechanism provides new insight into how cells eliminate damaged organelles. This mechanism may be relevant to kidney disease, where mitochondrial dysfunction and oxidative stress are common features of podocyte injury, diabetic kidney disease (DKD), and tubular damage.

Evidence from developmental models further supports a broader signaling role for migrasomes. In zebrafish embryos, migrasomes help regulate mesodermal cell migration by concentrating CXCL12, thereby influencing gastrulation and organ morphogenesis [31,42]. In addition, neutrophil-derived migrasomes have been linked to coagulation-related processes in experimental studies [43]. Although these findings are not kidney-specific, they suggest that migrasomes may participate in microenvironmental regulation in settings characterized by active cell migration and stress responses.

These properties provide a biological basis for investigating migrasomes in podocyte injury, inflammatory signaling, and metabolic stress in kidney disease.

## Isolation and experimental characterization of migrasomes

Migrasome identification requires more than recovery of extracellular particles from a biological fluid, because particle size or a single protein marker alone cannot establish migration-dependent biogenesis. In cell-based systems, the strongest evidence begins with direct visualization of vesicular swellings arising at the tips or branch points of retraction fibers behind migrating cells. Time-lapse fluorescence microscopy using TSPAN4-labeled cells, together with correlative light and electron microscopy, has shown that these structures emerge on retraction fibers as cells move forward and are released when the fibers break [15]. Thus, association with retraction fibers and demonstrable dependence on cell migration provide important biological criteria that distinguish bona fide migrasomes from particles classified only after isolation.

For physical isolation, differential centrifugation followed by density-gradient centrifugation has been widely used to enrich migrasomes from conditioned culture medium [15,44]. More recently, wheat germ agglutinin (WGA)-coupled magnetic beads combined with flow cytometry (WBFC) have enabled rapid capture and quantitative detection from culture medium, serum, and urine [44]. WGA recognizes glycosylated surface proteins and therefore is not intrinsically specific for migrasomes; specificity depends on subsequent detection of migrasome-associated surface proteins. Accordingly, WGA-based capture should be interpreted as an enrichment/capture strategy rather than independent proof of migrasome identity.

The clinical application of urinary migrasomes requires standardized pre-analytical processing. At present, no consensus protocol specifically addresses urinary migrasomes; therefore, existing migrasome studies should be combined with MISEV2023 and ISEV recommendations for urinary EVs. In the WBFC method, urine samples were centrifuged at 3000 × ***g*** for 10 min, followed by incubation with WGA-coupled magnetic beads for 1 h, and 1 ml urine at approximately 25°C was found suitable for quantitative analysis. For differential centrifugation, crude migrasomes can be enriched by 20 000 × ***g*** centrifugation, followed by PBS washing and, when higher purity is required, OptiPrep density-gradient purification [44]. To minimize technical variation, urine should preferably be processed within several hours after collection, kept at 4°C before processing, aliquoted before long-term storage, and stored at −80°C. Repeated freeze–thaw cycles should be avoided, ideally limiting samples to a single thaw. Basic urinary parameters, including pH, specific gravity, proteinuria, haematuria, leukocytes, and urinary creatinine, should be recorded because urine composition may influence vesicle recovery and downstream measurements. However, there is currently insufficient evidence to recommend routine adjustment of urine to a fixed pH before migrasome isolation. Future studies should systematically evaluate the effects of pre-clearing centrifugal force, storage duration, urine pH, and freeze–thaw cycles on TSPAN4^+^/ITGA5^+^ migrasome recovery. Until migrasome-specific guidelines are established, strict standardization and transparent reporting of all pre-analytical variables, in accordance with MISEV principles, are essential for improving reproducibility and comparability across studies.

Morphologically, migrasomes are relatively large membrane-bound structures, generally reported in the submicron-to-micron range (approximately 0.3–3 μm), although size varies with cell type and experimental context [27,41]. In migrating-cell preparations they characteristically appear as pomegranate-like or swollen structures on retraction fibers and can contain numerous smaller intraluminal vesicles [15]. In the urinary-migrasome assay developed by Yang et al., density-gradient-isolated migrasome standards from PAN-stimulated podocyte culture medium were predominantly oval particles of approximately 500 nm, and WGA-captured particles displayed a single limiting membrane by transmission electron microscopy (TEM) [44]. TEM therefore provides important structural evidence, but morphology alone is insufficient because isolated biofluid particles may lose the spatial information linking them to retraction fibers.

The migrasome limiting membrane is derived from the plasma membrane/retraction-fiber membrane rather than the endosomal multivesicular-body pathway characteristic of exosomes [15,35]. Migrasome formation is associated with specialized membrane organization involving sphingomyelin, cholesterol, integrins, and tetraspanin-enriched domains. TSPAN4 is the most widely used migrasome-associated marker, but a multi-marker strategy is preferable. Reported markers include TSPAN4, integrin α5 (ITGA5), NDST1, PIGK, CPQ, and EOGT [44,45]. In experimentally generated migrasome standards, these proteins are enriched, whereas canonical exosome-associated proteins such as CD9, CD63, and TSG101 are comparatively absent [44]. Nevertheless, no single marker should be regarded as absolutely migrasome-specific; orthogonal characterization using morphology, size, protein composition, and biological origin is therefore necessary.

A critical distinction should be made between migrasomes directly demonstrated in migrating cells and “migrasome-like” particles recovered from biofluids. In cell-based experiments, migration-dependent biogenesis can be supported by live-cell imaging, localization to retraction fibers, and experimental modulation of migration. For example, inhibition of podocyte migration reduces the migrasome signal, whereas high-glucose-induced migration increases it [44,46]. By contrast, particles isolated from urine or serum are spatially disconnected from the cells that produced them. Their classification therefore relies on convergent evidence such as size distribution, TEM morphology, enrichment of multiple migrasome-associated markers, depletion or assessment of markers of other EV populations, and—where possible—cell-of-origin markers. Yang et al. combined WGA capture with TSPAN4/ITGA5 detection and used synaptopodin to support a podocyte origin for urinary particles [44]. Importantly, urinary WGA-captured preparations can contain both migrasome- and exosome-associated proteins, illustrating the risk of co-isolation and the need for cautious terminology.

Accordingly, studies reporting “urinary migrasomes” should ideally document the isolation procedure and pre-analytical conditions; particle size and concentration; membrane morphology by TEM or another high-resolution imaging method; multiple positive migrasome-associated markers together with appropriate markers for potentially co-isolated EVs or cellular contaminants; and evidence supporting the proposed cell of origin. When direct evidence of retraction-fiber association or migration-dependent biogenesis is unavailable, particularly in biofluids, the designation should be interpreted operationally as particles with a migrasome-compatible phenotype rather than definitive proof that every detected particle arose through migrasome biogenesis. This distinction is central not only to methodological standardization but also to interpretation of the biological and biomarker evidence summarized in this review [42,44].

## Migrasomes in kidney diseases: integrated mechanisms and current evidence

Emerging evidence suggests that migrasomes contribute significantly to processes such as tumor progression [47,48], tissue regeneration [37], immune modulation [21,49], and wound healing [24] (Figure 3). Current kidney-focused studies have concentrated on podocyte-associated injury, membranous nephropathy, DKD, and renal cell carcinoma (RCC), with urine serving as a practical source for non-invasive detection (Tables 2 and 3).

**Figure 3:**
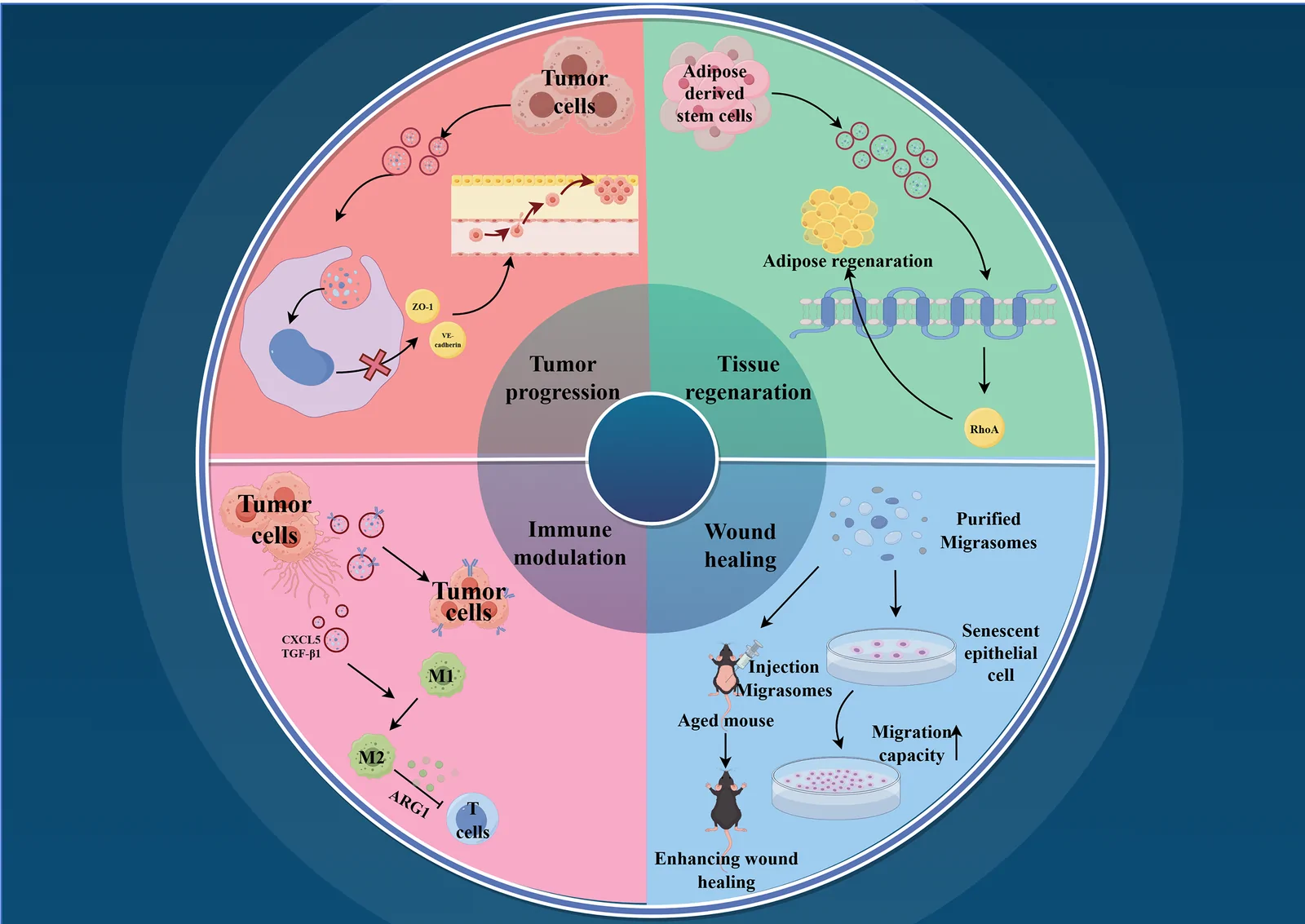
Clinical applications of migrasomes (**A**) Tumor progression: tumor cells can promote tumor metastasis by secreting migrasomes. (**B**) Tissue regeneration: migrasomes secreted by adipose-derived stem cells can promote fat regeneration. (**C**) Immune modulation: tumor cells can suppress the body’s immune response by secreting exosomes. (**D**) Wound healing. Figure created with Figdraw.

**Table 2:** Evidence on migrasomes in kidney diseases

Study	Year	Study design	Methods	Key findings
Liu et al. [46]	2020	*In vitro*, animal models, clinical urine samples	Podocyte injury (PAN/LPS/HG); migrasome assays; Rac1 pathway analysis; urinary quantification	1. Migrasome release increases during migration; enhancement is Rac1-dependent; 2. Urinary migrasomes increase under pathological injury; 3. Elevated podocyte-derived migrasomes in patients, supporting use as early noninvasive biomarkers of podocyte injury.
Yang et al. [44]	2024	Method development; clinical urine samples	WGA-coupled magnetic beads and flow cytometry (WBFC); detection of PLA2R on urinary migrasomes	1. WGA-coupled magnetic beads with flow cytometry (WBFC) isolated and quantified migrasomes from body fluids. 2. PLA2R was detected on urinary migrasomes from membranous nephropathy patients, enabling serum anti-PLA2R quantification to aid clinical diagnosis.
Shen et al. [68]	2025	Integrative bulk and single-cell transcriptomics with clinical data	Multi-omics integration; identification of migrasome-related lncRNAs; risk model construction; functional assays (FOXD2-AS1)	1. The model can predict kidney cancer risk and reveal its associations with immunity, mutations, and drug sensitivity. 2. FOXD2-AS1 significantly promotes renal cancer cell proliferation and migration.
Zhao et al. [69]	2025	Model development and validation in KIRC (ccRCC)	Survival modeling based on lncRNA expression; internal/external validation	1. Prognostic model of migrasome-associated lncRNAs established in ccRCC; 2. Predicts patient outcomes and supports risk stratification.

**Table 3:** Evidence supporting migrasome identity in kidney-related studies

Study	Disease/model	Evidence supporting migrasome identity	Morphology/EM	Multiple migrasome markers	Distinction from other EVs	Migration-dependent formation	Evidence level^*^
Liu et al. [45]	Cultured human/mouse podocytes; PAN nephropathy; patients with DN	Migrasomes isolated by sequential centrifugation; IF/imaging and particle characterization; urinary particles linked to podocyte injury	Yes	Limited relative to later studies	Yes	Yes	Strong
Yang et al. [43]	Urine from kidney-disease patients, including PMN	WBFC/WGA capture; TSPAN4/ITGA5 and podocyte-origin identification; migrasome standards characterized independently	Yes	Yes	Yes, but incomplete separation remains	Indirect for urinary particles;	Strong but not definitive for biofluid particles
Liu et al. [45]	HG-treated podocytes/DN patients	HG increased podocyte migrasome release and urinary migrasomes were elevated in DN	Yes	Limited	Yes	Yes	Strong experimental/moderate clinical
Jiao et al. [40]	Migrating cells; mitochondrial stress; neutrophils	Direct mechanistic demonstration of damaged mitochondria entering migrasomes and being eliminated by mitocytosis	Yes	Yes	No	Yes	Strong mechanistic evidence; non-renal
Liu et al. [61]	AGE-treated HUVECs	HUVEC migrasome formation and mitochondria-containing migrasome/mitocytosis assessed experimentally	Imaging evidence	Yes	Limited evidence for systematic exclusion of other EV classes	Migrasome/mitocytosis demonstrated in migrating endothelial-cell system	Moderate–strong mechanistic; non-renal
Chen et al. [65]	RCC cells, mouse models and RCC patients	ITM2B truncation promoted TSPAN4 recruitment, migrasome swelling/formation and caspase-7 migracytosis	Yes	Yes	No	Strong cellular evidence	Strong functional evidence
Shen et al. [66]	ccRCC TCGA + scRNA-seq + *in vitro* validation	Migrasome-associated lncRNA signature; knockdown of FOXD2-AS1 reduced expression of migrasome-marker proteins	No direct particle identification	Marker proteins assessed during validation	No	Cell migration assessed, but direct migrasome biogenesis was not established	Indirect/association evidence
Zhao et al. [67]	TCGA-KIRC	Migrasome-associated lncRNAs used to construct prognostic model	No	No direct particle characterization	No	No	Indirect bioinformatic evidence

### Podocyte and glomerular injury: migration-dependent release and injury biomarkers

Podocytes are essential components of the glomerular filtration barrier, and podocyte injury is a major contributor to proteinuria [50]. Canonical migrasome biogenesis is coupled to cell migration and retraction-fiber dynamics [20,35,51]. Quiescent podocytes exhibit limited migratory activity, whereas cellular injury can enhance migration, providing a mechanistic basis for increased migrasome formation and release [52]. This relationship is supported experimentally by Liu et al., who showed that lipopolysaccharide (LPS), puromycin aminonucleoside (PAN), and high glucose (HG) increased migrasome release from podocytes in a time- and dose-dependent manner [46]. The Ras-related C3 botulinum toxin substrate 1 (Rac1) signaling appears to participate in this response, because pharmacological Rac1 inhibition with EHT 1864 attenuated injury-induced migrasome release [46]. In a murine PAN nephropathy model, podocyte foot-process injury was evident by day 2, and urinary TSPAN4 increased from day 2, preceding detectable increases in serum creatinine and albuminuria on day 4 [46]. Clinical evidence is consistent with these observations: Yang et al. detected increased podocyte-derived urinary migrasomes in 288 patients with kidney diseases compared with 110 healthy controls using WGA-coupled magnetic beads and flow cytometry (WBFC) [44]. Thus, the migration dependence of migrasome biogenesis provides a mechanistic link between podocyte injury and urinary migrasome release, while the available animal and clinical data support their potential as non-invasive indicators of glomerular injury. However, urinary particles classified as migrasomes require careful experimental characterization, as discussed in Section 3.

### Membranous nephropathy: podocyte-derived migrasomes and PLA2R

Primary membranous nephropathy (PMN) is an autoimmune glomerular disease characterized by podocyte injury, with phospholipase A2 receptor (PLA2R) serving as a major target antigen [53]. Because migrasome formation increases with podocyte migration and injury, urinary podocyte-derived migrasomes may provide a compartment-linked readout of PMN. In urine samples from 79 patients with PMN, Yang et al. used WBFC to enumerate podocyte-derived migrasomes and found that PLA2R levels in the migrasome fraction were significantly higher than those in exosomes and positively correlated with serum PLA2R autoantibody titers [44]. These findings provide disease-specific clinical evidence that migrasomal cargo may reflect podocyte-associated antigenic processes. Nevertheless, urinary migrasome PLA2R should currently be regarded as a candidate complementary biomarker rather than a replacement for established serological or biopsy-based assessment, and prospective validation remains necessary.

### DKD: hyperglycemic stress and mitocytosis

DKD, a major microvascular complication of diabetes with worldwide prevalence, is a leading cause of chronic kidney disease [54]. The pathogenesis of DKD is complex and involves multiple interacting factors, including hemodynamic alterations [55], oxidative stress [56], and dysregulated lipid metabolism [57]. Direct evidence linking migrasomes to diabetic conditions remains limited but includes several experimental observations [58]. Migrasomes are specialized migration-dependent organelles, distinct in biogenesis from conventional EVs, that can transport signaling molecules such as CXCL12, facilitate long-range intercellular communication [34,37], and modulate the local microenvironment [24]. Cytokine signaling disturbance [59], abnormal endothelial–epithelial cross-talk [60], and ECM remodeling [61] are commonly observed in various renal lesions. Liu et al. demonstrated that high-glucose (HG) stimulation of cultured podocytes increased migrasome release, and urinary migrasome levels were higher in patients with DKD than in healthy controls. These findings support an association between hyperglycemic podocyte stress and migrasome release, as well as the potential biomarker value of urinary migrasomes, but they do not establish that migrasomes causally modify DKD progression [46]. A second potential mechanism is mitocytosis. Migrasome-mediated disposal of damaged mitochondria (mitocytosis) has been documented under conditions of reduced membrane potential and increased reactive oxygen species [40,41]. In human umbilical vein endothelial cells, advanced glycation end products induced mitochondrial depolarization, increased reactive oxygen species, and enhanced expulsion of damaged mitochondria through migrasome-associated mitocytosis [62,63]. This constitutes experimental evidence for a migrasome-dependent mitochondrial quality-control mechanism [40]. By contrast, a direct causal role for mitocytosis in kidney disease has not yet been established. Because mitochondrial dysfunction is a recognized feature of podocyte injury [64], DKD [65], and tubular dysfunction [66], it is biologically plausible that migrasome-mediated mitocytosis could influence renal metabolic stress; however, this remains an extrapolation from general migrasome biology rather than a kidney-specific demonstrated mechanism. Whether mitocytosis is protective, maladaptive, or context-dependent in renal disease therefore requires validation in kidney-specific *in vivo* models.

### RCC: migracytosis and tumor-microenvironment signaling

Among renal diseases, RCC provides comparatively direct evidence for a functional migrasome-mediated signaling pathway. Chen et al. reported that N-terminal truncation of integral membrane protein 2B (ITM2B) in RCC cells promotes migrasome formation and selective incorporation of active caspase-7. Macrophage uptake of these caspase-7-enriched migrasomes induced IL-6 secretion, which in turn promoted RCC-cell proliferation through a feedback loop [67]. Clinical specimens from 18 patients also showed higher truncated ITM2B levels in tumor tissue, and urinary migrasomes carrying truncated ITM2B were increased relative to healthy controls [67]. These findings directly connect migracytosis with macrophage-mediated tumor signaling in an RCC model. By contrast, broader claims that migrasomes globally remodel the renal tumor immune microenvironment, angiogenesis, or therapeutic response remain less well established. Transcriptomic studies have additionally identified migrasome-associated lncRNA signatures linked to prognosis, immune infiltration, tumor mutation burden, and drug sensitivity in clear cell RCC [68,69], but these associations should be distinguished from experimentally demonstrated migrasome-dependent mechanisms.

### Current evidence gaps in other kidney diseases

Direct evidence for migrasomes remains sparse in many major kidney diseases, including acute kidney injury, lupus nephritis, IgA nephropathy, tubulointerstitial disorders, and chronic fibrotic kidney disease. Rather than extrapolating uniformly from general migrasome biology, future studies may be prioritized according to disease features that are mechanistically aligned with established migrasome functions, particularly active cell migration, mitochondrial stress, immune-cell infiltration, and extracellular-matrix remodeling. A hypothesis-generating framework for such prioritization is summarized in Table 4. Importantly, these proposed priorities indicate biological plausibility and experimental tractability rather than established disease-specific roles for migrasomes.

**Table 4:** Proposed research-prioritization framework for migrasomes in understudied kidney diseases

Kidney disease	Mechanistic rationale	Priority research question	Key evidence gap	Priority
Acute kidney injury (AKI)	Cell migration/injury; mitochondrial stress; immune infiltration	Define tubular- and immune-cell migrasome sources; test effects on mitocytosis and early injury signaling.	No kidney-specific causal model demonstrating that migrasomes modify AKI severity or recovery.	High
Lupus nephritis (LN)	Immune infiltration; cytokine signaling; ECM remodeling	Map immune- and renal-cell migrasomes and test cargo-mediated immune-renal cross-talk.	No direct evidence linking migrasomes to immune-complex glomerular injury or treatment response.	High
IgA nephropathy (IgAN)	Immune inflammation; mesangial-podocyte communication	Characterize urinary/glomerular migrasomes and determine whether cargo reflects active inflammatory injury.	Disease-specific cellular sources, cargo, and causal functions remain undefined.	Moderate–high
Chronic fibrotic kidney disease	Cell migration; mitochondrial stress; ECM remodeling	Test fibroblast/tubular migrasomes in CXCL12-associated signaling, mitochondrial stress, and matrix-remodeling circuits.	Current rationale is largely extrapolated from non-renal wound-healing and general migrasome biology.	High
Tubulointerstitial disorders	Tubular injury; cell migration; immune infiltration; fibrosis	Use spatial imaging and lineage tracing to define migrasome release, recipient cells, and relation to fibrotic progression.	No established disease-specific migrasome pathway or validated urinary/tissue readout.	High
Focal segmental glomerulosclerosis (FSGS)	Podocyte injury/migration; mitochondrial stress	Test whether injury-induced podocyte migration drives migrasome release and whether urinary migrasomes track active injury.	Promising mechanistic fit, but direct FSGS-specific *in vivo* validation is lacking.	High

### Unresolved questions and research directions

Several proposed roles of migrasomes in kidney disease should currently be regarded as hypotheses rather than established mechanisms. First, although migrasome-mediated mitocytosis is experimentally established in migrating cells [40,41], its capacity to reduce oxidative stress or preserve renal function in DKD has not been demonstrated directly in kidney-specific animal models. Future studies should determine whether manipulating migrasome biogenesis or mitocytosis alters mitochondrial injury, albuminuria, histopathological damage, or renal function in diabetic models.

Second, the proposed contribution of migrasomes to renal inflammatory signaling and microenvironmental remodeling is supported mainly by their demonstrated ability in non-renal systems to transport proteins and RNAs and to enrich signaling molecules such as CXCL12, VEGFA, TNF-α, and IL-6 [34–38]. Whether these processes occur at functionally relevant levels in glomerular or tubulointerstitial compartments remains unresolved. Kidney-specific lineage-tracing, spatial imaging, cargo-profiling, and loss- or gain-of-function studies will be required to distinguish correlation from causation.

Third, evidence in renal malignancy is stronger for selected pathways but should not be generalized to all aspects of tumor-microenvironment remodeling. For example, RCC-derived migrasomes carrying active caspase-7 have been shown experimentally to induce macrophage IL-6 secretion and promote tumor-cell proliferation [67], whereas broader claims regarding immune remodeling, angiogenesis, or therapeutic targeting remain incompletely validated. Future work should define the disease stage, cellular source, recipient cell type, and cargo-specific effects of migrasomes and should validate these mechanisms in independent patient cohorts and *in vivo* models.

## Challenges and future directions

Migrasomes, as migration-dependent extracellular organelles that are mechanistically distinct from conventional EV subtypes, possess promising application prospects in the research field of kidney diseases (Figure 4). However, current research remains limited by several important challenges.

**Figure 4:**
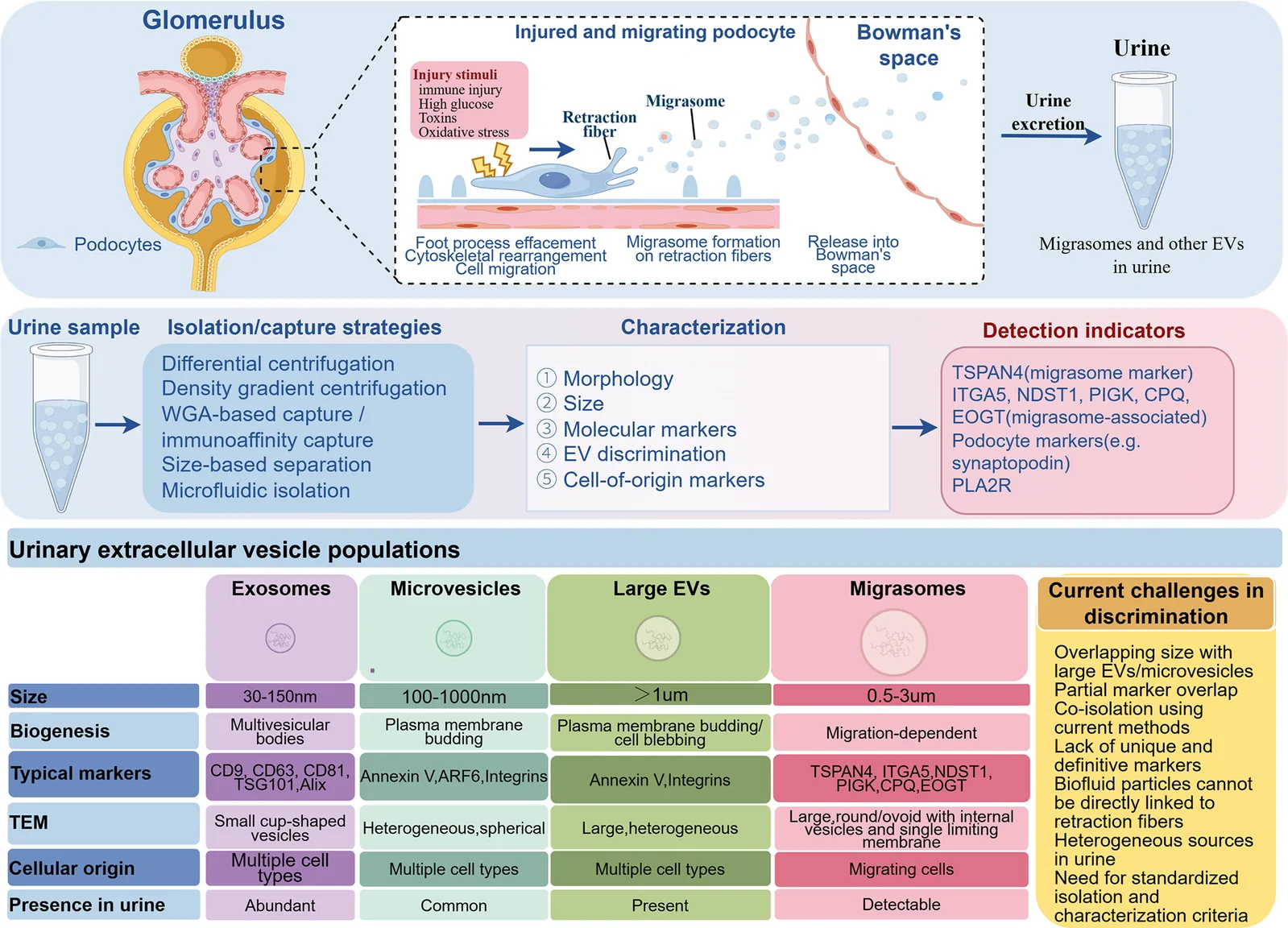
Proposed biogenesis, urinary detection, and characterization of podocyte-derived migrasomes in kidney disease Podocyte injury induced by immune-mediated injury, hyperglycemia, toxins, or oxidative stress may promote cytoskeletal rearrangement and cell migration. During migration, localized swellings form along retraction fibers and develop into migrasomes, which can subsequently detach and enter Bowman’s space and ultimately appear in urine. Urinary migrasomes can be enriched or captured using differential centrifugation, density-gradient centrifugation, lectin- or antibody-based capture, size-based separation, or microfluidic approaches. Their identification should rely on orthogonal characterization rather than a single marker, integrating particle morphology, size distribution, and multiple migrasome-associated proteins, including TSPAN4, ITGA5, NDST1, PIGK, CPQ, and EOGT. Cell-of-origin markers such as synaptopodin may further support a podocyte origin, whereas disease-associated cargo such as PLA2R may provide additional information in specific diseases, including PMN. The lower panel illustrates the major challenge in interpreting urinary migrasome measurements. Migrasomes overlap in size and, potentially, molecular composition with other urinary EV populations, particularly large EVs and microvesicles. In contrast with exosomes derived from the endosomal multivesicular-body pathway and microvesicles generated by plasma-membrane budding, bona fide migrasomes are defined by their migration-dependent formation on retraction fibers. However, this spatial and biogenetic information is lost after particles are released into biofluids. Therefore, urinary particles displaying a migrasome-compatible phenotype should ideally be characterized using multiple complementary criteria, including morphology, size, migrasome-associated markers, cell-of-origin markers, and assessment of potentially co-isolated extracellular-vesicle populations. The figure highlights both the potential of urinary podocyte-derived migrasomes as non-invasive biomarkers of kidney injury and the current need for standardized criteria that reliably distinguish them from other urinary EVs. Figure created with Figdraw.

Although migrasomes show great promise in kidney disease research, several challenges remain. First, the mechanisms underlying migrasome biogenesis, molecular composition, and physiological functions are not yet fully understood. While preliminary studies suggest migrasomes may play a role in podocyte injury, more systematic investigation is needed to clarify their precise roles in kidney disease pathogenesis [70]. Additionally, their specificity, sensitivity, and diagnostic value for distinguishing among different kidney diseases must be validated through large-scale clinical studies [23].

Second, while methods for isolating migrasomes—such as differential centrifugation and WBFC—have been developed, there is still a pressing need to standardize these techniques for consistency across research groups. Advances in purification technologies, as well as the identification of specific migrasome markers, are crucial for reliable detection. To ensure reproducibility and accuracy, standard protocols for sample collection, storage, and processing must be established before clinical adoption [71]. Moreover, targeted therapeutic strategies, including modulating migrasome biogenesis or utilizing them as delivery vehicles, require more research to optimize delivery, safety, and scalability [27,72].

Opportunities include early injury detection, disease-linked cargo readouts (e.g. PLA2R [57]), and serial monitoring. However, their clinical application remains limited by methodological variability and insufficient large-scale validation. The clinical value of transferable entities ultimately depends on the reproducibility of the detection methods and the establishment of a disease-specific interpretation system. Therapeutic applications of migrasomes are at a substantially earlier stage than their diagnostic investigation and should currently be considered conceptual or preclinical rather than clinically actionable. Potential strategies include suppressing pathological migrasome biogenesis or cargo transfer in settings where migrasomes contribute to disease progression, as well as exploiting their capacity for localized cargo delivery [25,38,70]. Nevertheless, these approaches have not been adequately validated in kidney-specific therapeutic models. Before clinical translation, substantial foundational work is required to define the biodistribution, tissue tropism, circulation and clearance kinetics, cellular uptake, and off-target accumulation of endogenous or engineered migrasomes. Equally important are systematic safety assessments addressing immunogenicity, unintended intercellular signaling, potential pro-inflammatory or pro-tumor effects, dose-related toxicity, and the consequences of interfering with physiological migrasome functions. Therapeutic efficacy must also be demonstrated in appropriate *in vivo* kidney-disease models using clinically relevant endpoints, followed by evaluation of dose-response relationships, reproducibility, manufacturing scalability, cargo-loading stability, and batch-to-batch quality control. Thus, although migrasome-based intervention represents an intriguing therapeutic direction, current evidence is insufficient to support clinical application, and its feasibility will depend on rigorous preclinical characterization of biodistribution, safety, and efficacy.

Future research on migrasomes in nephrology should improve migrasome isolation and detection with multi-omics approaches [21,39]; delineate their pathophysiological roles in kidney diseases such as DKD and focal segmental glomerulosclerosis, with particular attention to podocyte injury, inflammation, and fibrosis [73]; evaluate their utility as liquid-biopsy biomarkers for disease staging and prognosis [44]; and develop therapeutic strategies that target migrasome-mediated intercellular communication [39]. These efforts must address major technical challenges, including the marked heterogeneity of migrasomes and their complex dynamic behavior *in vivo* [27]. Furthermore, the clinical translational potential of migrasomes needs to be validated through multicenter collaborative studies [51].

## Conclusion

In summary, migrasomes are associated with renal pathological processes, including podocyte injury and membranous nephropathy, suggesting their potential diagnostic value. Migrasomes may also be involved in renal tumor progression and in remodeling of the renal microenvironment. However, their roles and underlying mechanisms in other renal diseases are still not well defined. Further studies are needed to determine whether migrasomes can be used for early diagnosis, disease monitoring, or therapeutic development in kidney diseases.

## Perspectives

- Migrasomes are migration-dependent extracellular organelles formed on retraction fibers and are mechanistically distinct from conventional EV subtypes such as exosomes, microvesicles, and apoptotic bodies; they participate in intercellular signaling, cellular-material disposal, and mitochondrial quality control.
- This review focuses on the biological characteristics of migrasomes most relevant to kidney disease, summarizes the current evidence in the field of nephrology, and explores the potential of migrasomes in diagnostics and translational medicine, as well as the major obstacles to their clinical application.
- The roles and underlying mechanisms in other renal diseases are still not well defined. Further studies are needed to determine whether migrasomes can be used for early diagnosis, disease monitoring, or therapeutic development in kidney diseases.

## Open Access

Open access for this article is provided via our Subscribe to Open programme, made possible through the support of our subscribing institutions. Learn more here: https://portlandpress.com/pages/open_access_options_and_prices#conditional.

## Abbreviations

AKI: acute kidney injury
ccRCC: clear cell renal cell carcinoma
CPQ: carboxypeptidase Q
CXCL12: C-X-C motif chemokine ligand 12
DKD: diabetic kidney disease
ECM: extracellular matrix
eGFR: estimated glomerular filtration rate
EOGT: EGF domain-specific O-linked N-acetylglucosamine transferase
EVs: extracellular vesicles
FSGS: focal segmental glomerulosclerosis
GBM: glomerular basement membrane
HG: high glucose
IgAN: IgA nephropathy
IL-6: interleukin-6
ITGA5: integrin subunit alpha 5
ITM2B: integral membrane protein 2B
LN: lupus nephritis
lncRNA: long non-coding RNA
LPS: lipopolysaccharide
NDST1: N-deacetylase and N-sulfotransferase 1
NTA: nanoparticle tracking analysis
PAN: puromycin aminonucleoside
PIGK: phosphatidylinositol glycan anchor biosynthesis class K
PIP5K: phosphatidylinositol 4-phosphate 5-kinase
PLA2R: phospholipase A2 receptor
PMN: primary membranous nephropathy
PTEN: phosphatase and tensin homolog
Rac1: Ras-related C3 botulinum toxin substrate 1
RCC: renal cell carcinoma
SEC: size-exclusion chromatography
SMSs: sphingomyelin synthases
TEMs: tetraspanin-enriched microdomains
TNF-α: tumor necrosis factor-α
TSPAN4: Tetraspanin 4
VEGFA: vascular endothelial growth factor A
WBFC: WGA-coupled magnetic beads and flow cytometry
WGA: wheat germ agglutinin
